# The Hammersmith Meeting: An Impracticable Programme

**Published:** 1913-12-27

**Authors:** 


### THE HAMMERSMITH MEETING.

### An Impracticable Programme.

Elsewhere we publish a verbatim report of the Hammersmith meeting of the National Medical Guild on the 19th inst. The Hospital gave publicity to the objects of the Guild and the views of its promoters during several successive weeks, but the profession were found to dislike the idea of a trades union, and the plan was consequently suspended for a time. The Hammersmith meeting indicates that a new and energetic attempt is to be made to put the Medical Guild on its feet, providing, as Dr. Townsend's resolution states, "that the Guild by using every legitimate means can compel the British Medical Association to recognise the Guild as an auxiliary body."

### An Ironclad Fighting Fund.

The avowed object and use of the Guild as explained by Mr. Turner and the Guild's Secretary, Dr. Raiment, are declared to be that, unlike the British Medical Association, it can raise an ample defence or guarantee fund by compulsory levy upon its members to a maximum of £20 per head per year, and hold it for the protection of medical practitioners, because the Judges could not touch it, under the Trades Unions laws. The claim is therefore that the Guild will provide an Ironclad Fighting Fund to sufficiency. Dr. Raiment estimates that £200,000 can be raised by levies on its members by the end of "three peaceful years." But Mr. E. B. Turner in his speech did not accept this, for he declares that £137,000 was not enough when the British Medical Association raised it last year, in a few months. The difference between the two sums mentioned is not great, and the profession and practitioners generally will naturally ask, what stronger position would the Medical Guild be in, in three years, with £200,000—Mr. Turner requires this Fund in less than two years—than the British Medical Association was in with its guarantee fund of £137,000 last year? The kernel of the whole matter is what capital sum will in fact create a fighting fund large enough to enable practitioners to defend themselves against the politicians? £500,000 is held to be the minimum amount for such a fund, if it is to be effective when the hour of battle—*strikes*. Now if Dr. Raiment's estimate be correct it would take the Guild, if it succeeded in enrolling the whole body of medical practitioners in the country, seven and a-half years to raise this half million for a fighting fund. During those seven and a-half years every practitioner who joins the Guild as a member must be compelled to put up £20 each year of his own money, or £150 in the whole, to produce the £500,000. After all, finance is the foundation of every strong enterprise; but the financial scheme propounded at the meeting on the 19th inst., on examination would appear to be impossible of achievement. We fear therefore, as it stands, it is not one which can be seriously entertained by members of the profession who understand business. Is there any practical answer to be urged against this conclusion which is forced upon us by the statements made at the meeting?

### Standards of Conduct.

Again, what the profession has to keep constantly in mind at the present moment is that there are matters of infinitely greater importance, even from the point of view of defence and a fighting policy, than the monetary question. At this crisis the profession of medicine needs the help of every practitioner who has a high standard of personal honour, who will not consent to occupy a position as a mere tradesman, because he feels that he owes it to his university, his medical school; his profession, and himself that he shall do nothing to lessen his authority as a member of a learned profession. That this position is one which is being recognised throughout the country with gratitude by insured persons who belong to the more intelligent and thoughtful members of the working and other industrial portions of the community has been made incontestably plain. We published in The Hospital, December 28, 1912, page 345, a striking statement by a working-man of the foreman type. It proceeds:

"What sort of doctoring are we likely to get under the Act? As a general rule it will be the unsuccessful and the young beginner who will offer himself for the work, and things will be worse as time goes on. An inferior kind of man will be attracted to the profession, the sort of man who will be contented with small fees. The workman can't go and sit in the doctor's waiting room amongst a crowd of other patients to wait his turn; he can't afford the time. So it means that your 35s. and 40s. a week man will decide to pay twice. He will pay the insurance and have his own doctor." A truly remarkable forecast seeing that this workman's view is becoming the practice of an ever-increasing number of insured persons, Mr. E. B. Turner, in the early part of his speech, insisted that "the whole of the relations of the medical profession and those of their patients of the industrial classes were going to be cast into the melting pot." He explained his meaning to be that the medical attendance of all uninsured persons, including the dependants of those at present insured, would by the politicians be placed soon under the care of a panel doctor. Mr. Turner appears not to have grasped the attitude of the working classes who lead the opinion of their mates on this question. Has he ever read the signs of the times? For instance, that the Leader of the Opposition has declared himself in favour of abolishing compulsion on the employed to pay for National Insurance, the facts we have adduced, and the present position of political parties? We think not, for it would seem infinitely more probable that panel practice will received a most damaging blow, and that working-class opinion will secure to every free man and woman the right to be attended by his or her own doctor. If this be so it is not the panel doctors who will have behind them the goodwill and support of the majority of the 37,000,000 who may speedily enjoy the benefits of National Insurance, but the non-panelite practitioners. Why? Because our industrial classes recognise a man and a gentleman, and love him before all other men with whom they have to deal in ordinary life.

### Will Mr. Turner Kindly Explain?

We should like to have Mr. Turner's answer to this criticism, because as the opinion of insured persons, or a majority of them, is to-day, there can be no question that if the non-panel practitioners throughout the country combine speedily, by becoming members of the National Medical Union, when their names are published, as we hope to publish them in the near future, many of the most far-seeing and best of the practitioners, who were forced on the panel in the early part of the present year, will retire most certainly from the panel and join the National Medical Union. One factor must be definitely ascertained before a final judgment can be uttered on some points in the present controversy. That factor is the effect which recent changes in the British Medical Association may have upon the membership of that body. If, as is held by medical opinion in many districts of the United Kingdom, at least 10,000 members retire from the British Medical Association, the non-panelite policy will triumph, and the medical profession regain its authority with the public.

